# Planned vs. Performed Treatment Regimens in Diabetic Macular Edema: Real-World Evidence from the PACIFIC Study

**DOI:** 10.3390/jcm14093120

**Published:** 2025-04-30

**Authors:** Christos Haritoglou, Matthias Iwersen, Bettina Müller, Erik Beeke, Hüsnü Berk, Matthias Grüb, Katrin Lorenz, Martin Scheffler, Focke Ziemssen

**Affiliations:** 1 Augenklinik Herzog Carl Theodor, Nymphenburger Str. 43, 80335 München, Germany; 2 Novartis Pharma GmbH, Roonstr. 25, 90429 Nürnberg, Germanybettina.mueller@novartis.com (B.M.); 3 Visualeins MVZ für Augenheilkunde und Anästhesie GmbH, Am Finkenhügel 7 B, 49076 Osnabrück, Germany; 4 St. Elisabeth-Krankenhaus GmbH, Klinik für Augenheilkunde, Werthmannstr. 1, 50935 Köln, Germany; 5 Matthias Grüb & Kollegen, Bahnhofstr. 7-9, 79206 Breisach am Rhein, Germany; matthias.grueb@grueb.eu; 6 Augenklinik und Poliklinik der Universitätsmedizin Mainz, Langenbeckstr. 1, 55131 Mainz, Germany; 7 Augenheilkunde Rhauderfehn, Dr. med. Martin Scheffler, Rhauderwieke 3, 26817 Rhauderfehn, Germany; 8 Klinik für Augenheilkunde, Universitätsklinikum Leipzig, Liebigstraße 12, 04103 Leipzig, Germany; 9 Department für Augenheilkunde, Eberhard-Karls-Universität Tübingen, Elfriede-Aulhorn-Str. 7, 72076 Tübingen, Germany

**Keywords:** diabetic macular edema (DME), vascular endothelial growth factor (VEGF) inhibitors, real-world evidence, treatment adherence, intravitreal injections, observational study, treatment strategies, clinical guidelines, treatment deviations, patient-centered care

## Abstract

**Background:** Intravitreal injections of vascular endothelial growth factor (VEGF) inhibitors are standard for diabetic macular edema (DME), yet a gap exists between clinical guidelines and actual practices. This study aimed to investigate the extent of deviation between physician-planned and actually performed treatment regimens. **Methods:** The PACIFIC study (NCT04847895) was a prospective, multicenter, non-interventional study conducted in Germany, the Netherlands, and Switzerland. A total of 910 patients with DME receiving ranibizumab were enrolled. Physicians documented the intended treatment regimen at baseline, and actual treatment patterns were retrospectively derived from the timing of visits and injections over a 24-month observation period. **Results:** Although most physicians initially planned fixed or pro re nata (PRN) regimens, 77% of pretreated and 73% of treatment-naïve patients ultimately followed a monitor and extend strategy. Treatment discontinuation was frequent (58.8% and 59.4%, respectively), and injection frequencies remained below recommended levels, although central retinal thickness improved over time. **Conclusions:** The study highlights a consistent and clinically relevant discrepancy between planned and actual treatment delivery in DME care, underscoring the need for better adherence to guideline-informed strategies in routine practice.

## 1. Introduction

Intravitreal injections of vascular endothelial growth factor (VEGF) inhibitors, such as ranibizumab, represent the standard of care for diabetic macular edema (DME). Various clinical trials have demonstrated the efficacy and safety of ranibizumab in treating DME [1,2,3,4]. In addition, researchers have published several studies demonstrating the effectiveness of ranibizumab in treating DME in a real-world setting. However, these studies have methodological limitations; for instance, previous studies did not compare the initially planned treatment regimen to the one actually performed [5,6,7,8]. In particular, the number of treatments was low in previous retrospective cohorts. According to the observational study design, there were several possible explanations: either only a few injections were initially agreed upon, a conscious or unconscious decision was made against further injections, or early withdrawal from longer follow-up distorted the figures.

Since clinicians often observe continuous but slow improvement under initial treatment and macular fluid needs time to decline [9], guidelines recommend intensive treatment, e.g., providing an initial series of injections, followed by further injections as needed (“pro re nata”) until maximum visual acuity is achieved (German guidelines [10]). Retina specialists consider the condition to be stable when the patient’s visual acuity remains unchanged and/or no signs of disease activity are detected during consecutive monthly tests. They monitor these parameters by assessing best corrected visual acuity (BCVA) and using optical coherence tomography (OCT) [11]. The initial intensity has been shown to be important for later outcomes [12,13,14]. After the first 6 months, a “treat and extend” regimen might be an excellent alternative to the “pro re nata” regimen, particularly if only one eye is affected [10].

The poorer outcome of crossover groups with late treatment started proved the impact on therapy outcomes [15]. Visual acuity at the start of therapy is an important prognostic factor for the level of visual acuity that can be reached by treatment [16,17,18,19,20,21]. Clearly, relatively good visual acuity at baseline limits “room for improvement” in terms of gains in letters. However, despite lower relative gains in letters, better absolute visual acuity can be achieved in these patients, and high visual acuity can be maintained in comparison to starting treatment with low visual acuity. Thus, the aim should be to diagnose DME as early as possible and initiate treatment immediately [22].

At the time of planning the PACIFIC study, no data regarding the efficacy of different treatment regimens used in routine clinical practice had been available. Thus, the PACIFIC study was conducted to determine the utilization and effectiveness of these various treatment regimens in routine clinical practice and their dependence on the application of different treatment regimens.

## 2. Materials and Methods

### 2.1. Study Design

The PACIFIC study was an observational, multicenter, open-label, single-arm study of patients with DME in Germany, the Netherlands, and Switzerland who were treated with ranibizumab.

Patient enrollment started in June 2015 and ended in March 2019. A minimum of one follow-up visit per year was required to maintain patient participation in the study over the prospective observational period of 24 months. This was a non-interventional study (NIS) in which no therapy, diagnostic/therapeutic procedure, or visit schedule was imposed.

### 2.2. Subjects

Patients could be included in the study if they were being treated with ranibizumab for any approved indication and gave written informed consent. Treatment-naïve (untreated) and pretreated patients were included. Pretreatment was defined as prior treatment with ranibizumab, other anti-VEGF drugs, intravitreal corticosteroids, photodynamic therapy, or laser coagulation before the start of the PACIFIC study (baseline). A total of 185 sites participated in the PACIFIC study, with 4948 patients in the safety evaluation set (SES) and 4932 patients in the full analysis set (FAS) for all indications. Of these, 913 patients in the SES cohort and 910 patients in the FAS had DME and were the subject of the present evaluation.

### 2.3. Statistical Methods

In the present analysis, descriptive summary statistics were calculated for the data from all the DME patients. Patient characteristics were documented at baseline. Variables such as details of ranibizumab injections and planned treatment regimens, BCVA examinations, OCT examinations, and adverse events (AEs) were documented at each visit. Diseases from medical history and AEs were coded using the Medical Dictionary for Regulatory Activities (MedDRA) version 24.0. For variables with predictive validity, appropriate strata were created. Statistics were calculated using the SAS software version 9.4 (SAS Institute, Cary, NC, USA). All safety-relevant analyses were performed for the SES, and all analyses relevant for utilization and effectiveness were performed for the FAS. Analyses were performed according to a predefined analysis plan.

The intended treatment regimens were documented by the treating physicians. The actual treatment regimens used were derived from the documented time points. Treatment regimens were defined as follows: “fixed” scheme, with regular intervals between follow-up visits and reinjection at every visit; “pro re nata”, with regular intervals between follow-up visits and reinjection as needed; “treat and extend”, with intervals between follow-up visits extended step by step and reinjection at every visit; and “monitor and extend”, with variable intervals between follow-up visits and reinjection as needed.

## 3. Results

### 3.1. Participants

A total of 913 patients with DME (in the SES) were enrolled in the PACIFIC study, and 910 patients were included in the FAS. Among those, 458 patients had previously received treatment, and 452 patients were treatment naïve. Most patients were from Germany (423 pretreated, 433 naïve), and few were from the Netherlands (19 pretreated, 0 naïve) or Switzerland (16 pretreated, 19 naïve). Among patients from Germany, the vast majority had public health insurance (92.0% of pretreated patients, 93.3% of treatment-naïve patients).

Demographics and baseline disease characteristics are shown in Table 1. Slightly more patients in the PACIFIC study were male, and the mean age was approximately 66 years for both pretreated and treatment-naïve patients. The mean baseline HbA1c value was approximately 52 mmol/mol for pretreated patients and 49 mmol/mol for treatment-naïve patients. The mean baseline BCVA, measured as early treatment diabetic retinopathy study (ETDRS) letters, was 66.3 of the ETDRS letters for pretreated patients compared to 64.1 of the ETDRS letters for treatment-naïve patients. Most patients had a baseline OCT examination, with a mean central retinal thickness of 330.0 µm for pretreated patients and 352.8 µm for treatment-naïve patients. The documented fundus lesions did not differ between the naïve and pretreated study participants (Appendix A, Table A1).

### 3.2. Treatments

Pretreated patients received a mean (±SD) number of 5.8 ± 2.9 injections (*n* = 266) of ranibizumab for the study eye in the first year (among those patients with documentation for at least the first year) and 10.6 ± 5.4 injections (*n* = 55) in two years (among those patients with documentation for at least two years). Treatment-naïve patients with DME received 6.4 ± 2.8 injections (*n* = 264) in the first year and 9.2 ± 4.5 injections (*n* = 52) in two years.

In the first 6 months, the pretreated patients had a mean (±SD) of 3.5 ± 1.6 injections. In the first 6 months, treatment-naïve patients had a mean (±SD) of 4.1 ± 1.5 injections.

The median time between the baseline BCVA examination and the first ranibizumab injection was 6 days (mean ± SD: 12.6 ± 17.1 days; *n* = 386) for pretreated patients with DME and 5 days (mean ± SD: 10.7 ± 15.1 days; *n* = 408) for treatment-naïve patients. The median time between subsequent study eye injections was 44.4 days (mean ± SD: 50.8 ± 23.1 days; *n* = 398) in pretreated patients and 39.2 days (mean ± SD: 45.1 ± 19.9 days; *n* = 432) in treatment-naïve patients.

**Figure 1:**
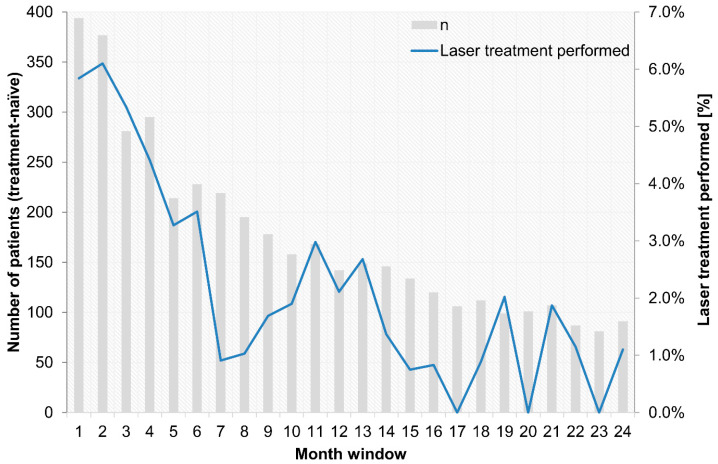
Concomitant laser treatment over the 24-month observational period for treatment-naïve patients.

**Figure A1:**
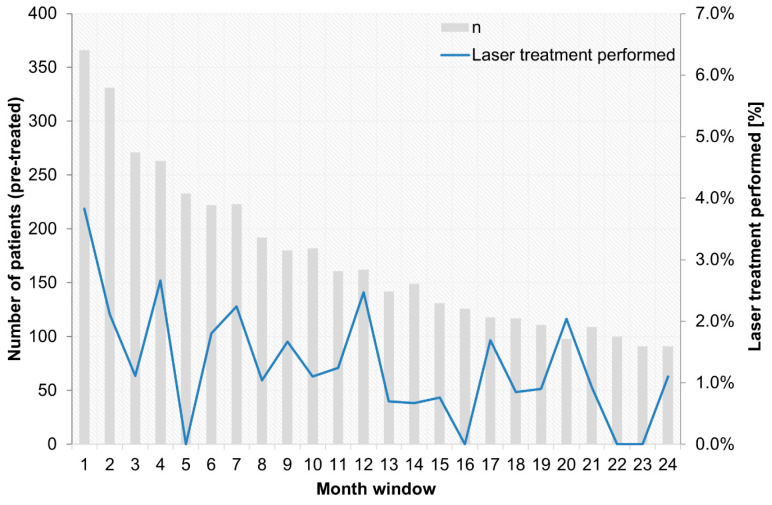
Concomitant laser treatment over the 24-month observational period for pre-treated patients.

**Figure A2:**
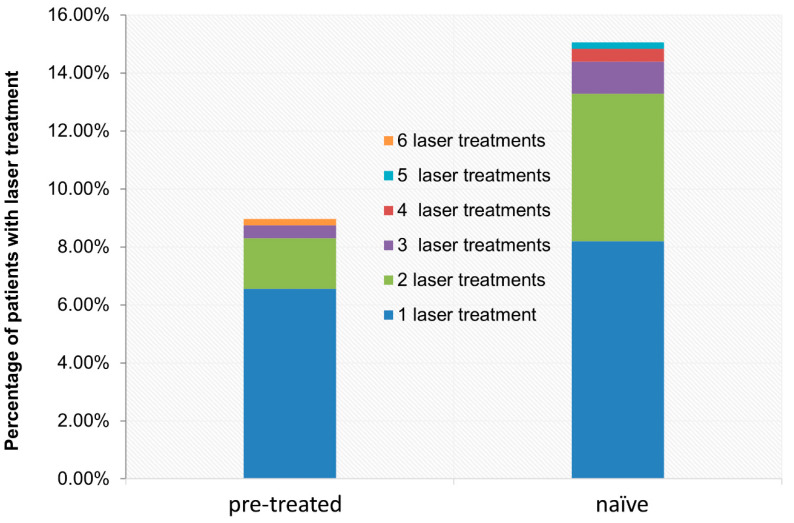
Cumulative percentages of concomitant laser treatments for pre-treated and treatment-naïve patients.

The course of concomitant laser treatments over the observational period for treatment-naïve patients is shown in Figure 1. Concomitant laser treatments for pretreated patients are provided in Appendix A, Figure A1, and a cumulative depiction of treatment-naïve and pretreated patients is provided in Appendix A, Figure A2. Most patients who received concomitant laser treatments had at least one laser treatment within the first 6 months. In total, 9.0% of the pretreated patients and 15.1% of the treatment-naïve patients received concomitant laser treatments during the observational period.

The pretreated patients remained in the documented study treatment for a median follow-up of 442 days, i.e., approximately 14.5 months, and treatment-naïve patients for a median of 453 days, i.e., approximately 15 months. Premature discontinuation of the study was documented for 258 of the 439 pretreated patients (58.8%) and for 252 of the 424 treatment-naïve patients (59.4%). The percentage of patients who prematurely discontinued the study was slightly greater among patients with renal insufficiency (68.0% of 25 pretreated patients, 88.9% of 18 treatment-naïve patients) and among patients with HbA1c >9% (66.7% of 21 pretreated patients, 68.4% of 19 treatment-naïve patients).

### 3.3. Treatment Regimens

**Figure 2:**
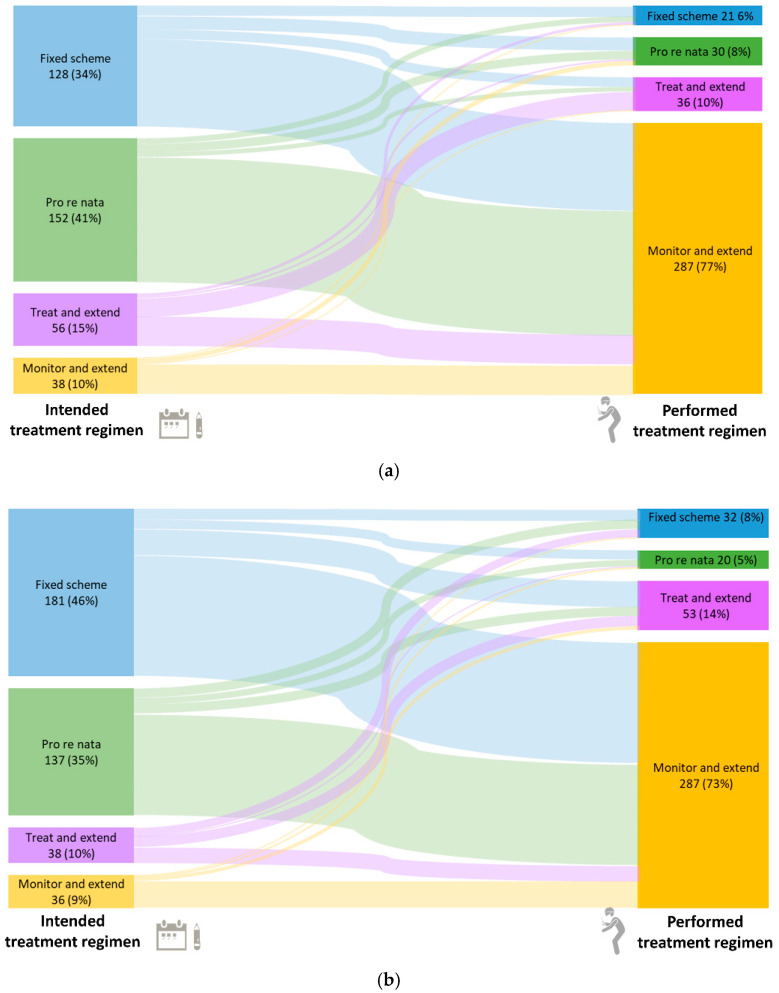
Intended treatment regimens at baseline and actual treatment regimens in the last month for pretreated (**a**) and treatment-naïve (**b**) patients. Percentages are based on non-missing observations. In total, 443 pretreated patients had observations in the last month. Information on the treatment regimen was missing for 26 patients, and for 43 patients, the intended treatment regimen was changed during the study; therefore, these patients are not included in this figure. Of the 443 naïve patients with observations in the last month, 16 patients were missing information, and the intended treatment regimen was changed for 35 patients.

**Figure A3:**
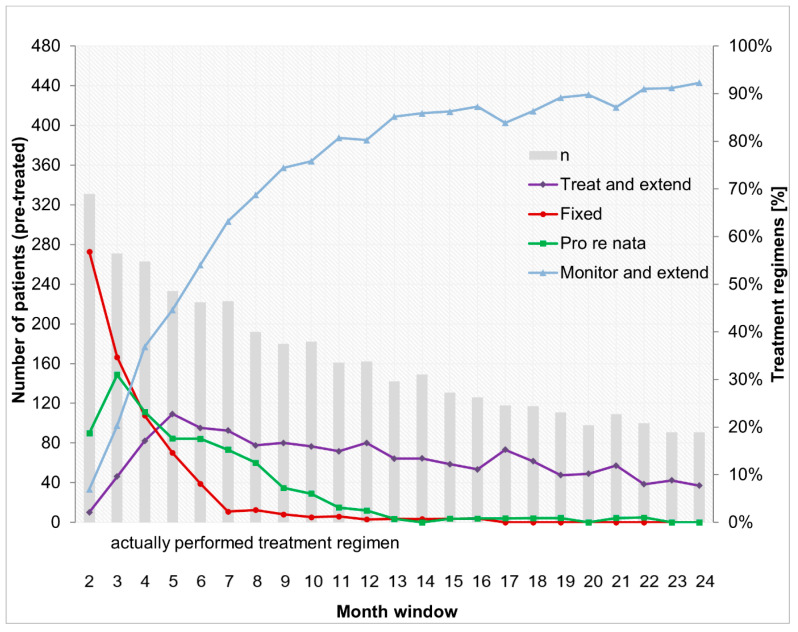
Actually performed treatment regimens over the 24-month observational period for pre-treated patients.

**Figure A4:**
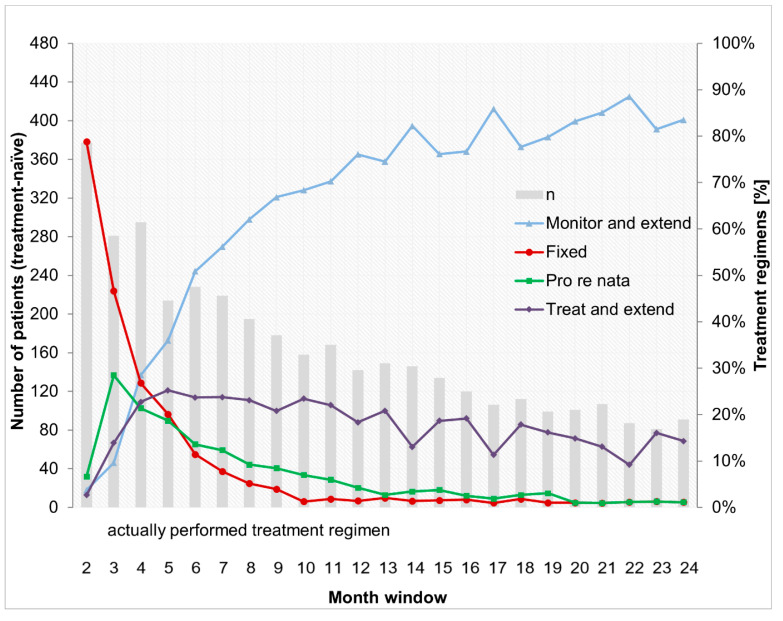
Actually performed treatment regimens over the 24-month observational period for treatment-naïve patients.

The intended treatment regimens, as documented at baseline, as well as the actually performed treatment regimens in the last month, based on statistical derivation from temporal patterns of visits and injections, are shown in Figure 2. At baseline, a “fixed” scheme and “pro re nata” were the most commonly used treatment regimens. For pretreated patients, “pro re nata” was slightly more frequent (41% “pro re nata”, 34% “fixed”), while for treatment-naïve patients, a “fixed” scheme was more frequent (46% “fixed”, 35% “pro re nata”). However, overall, the treatment regimen used was “monitor and extend” for the majority of patients (77% of pretreated patients and 73% of treatment-naïve patients). The time-dependent trends in treatment regimens over 24 months are shown in Appendix A, Figure A3 and Figure A4 for pretreated and treatment-naïve patients, respectively.

### 3.4. Visual Acuity

**Figure 3:**
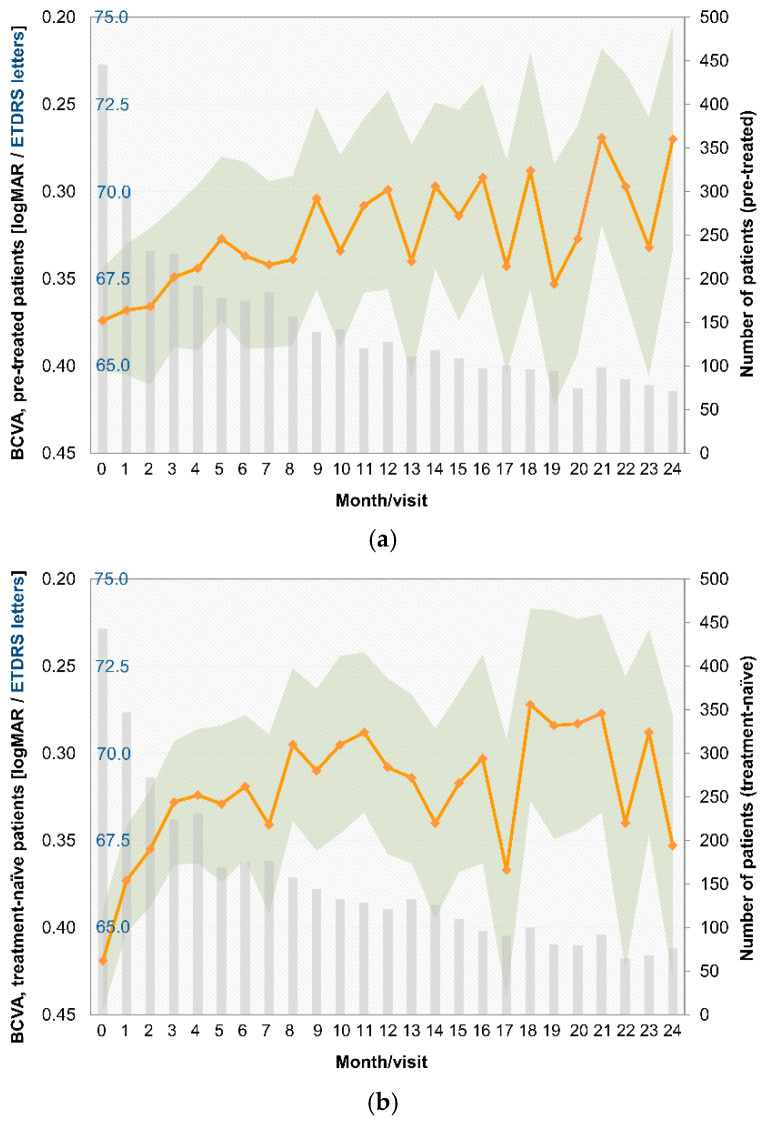
BCVA by month, logMAR, and ETDRS values for pretreated (**a**) and treatment-naïve patients (**b**).

At the end of the observational period (month 24), the mean (±SD) BCVA was logMAR 0.270 ± 0.274 or 71.5 ± 13.7 of ETDRS letters (n = 71) for pretreated patients and logMAR 0.353 ± 0.330 or 67.3 ± 16.5 of ETDRS letters (n = 76) for treatment-naïve patients. Compared to the baseline BCVA, the BCVA of the study eye improved during each month of the study. The changes from baseline for pretreated and treatment-naïve patients are shown in Figure 3.

**Figure A5:**
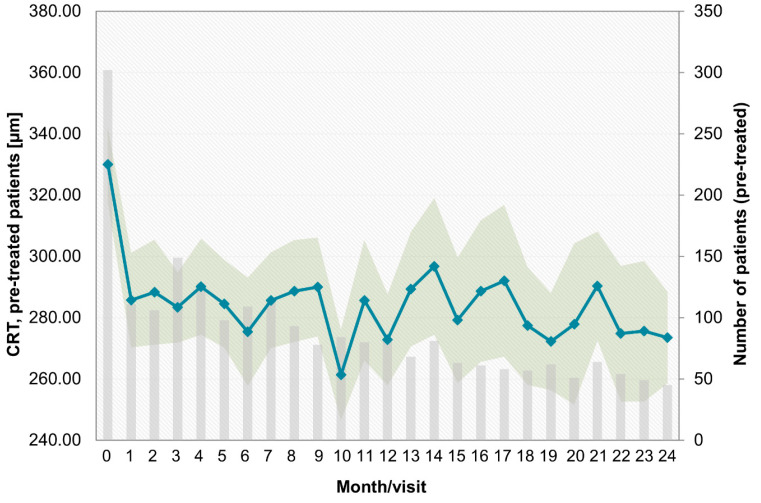
Central retinal thickness (CRT) by month for pre-treated patients.

**Figure A6:**
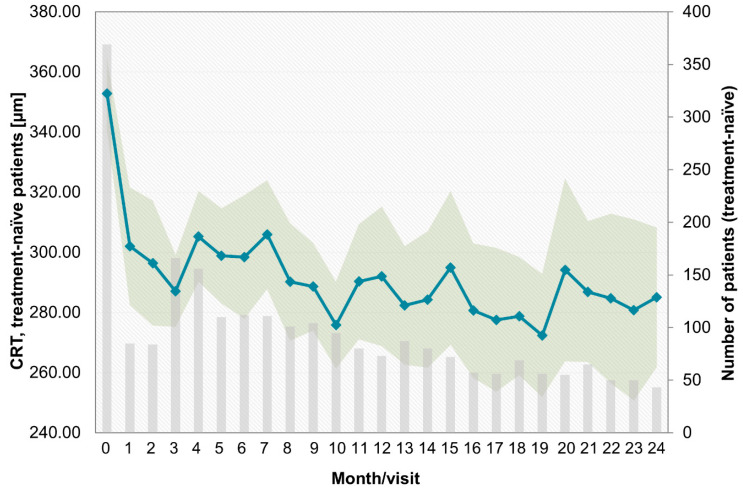
Central retinal thickness (CRT) by month for treatment-naïve patients.

Pretreated patients received a total mean (±SD) number of 9.2 ± 5.2 OCT examinations of the study eye (n = 55), and treatment-naïve patients received 8.6 ± 5.7 OCT examinations (n = 52, among patients with documentation for at least two years). Decreases in the central retinal thickness of the study eye were observed at all visits compared to baseline. The course of central retinal thickness by study month is provided in Appendix A, Figure A5 for pretreated patients and in Appendix A, Figure A6 for treatment-naïve patients.

### 3.5. Adverse Events

Among pretreated patients, 41.2% (of 459 patients in the SES) experienced AEs. Severe AEs (SAEs) were reported for 27.7%, and serious adverse drug reactions (SADRs) were reported for 6.1%. The most frequently reported AEs were diabetic retinal edema (5.0%), cystoid macular edema (4.6%), retinal edema (4.1%), and concomitant disease aggravation (4.1%). Of a total of 551 AEs, 33.6% had mild intensity, 40.1% had moderate intensity, and 16.3% had severe intensity. The incidence of AEs was greater among pretreated patients with an HbA1c >9% (66.7% of 21 patients).

Among treatment-naïve patients, 33.5% (of 454 patients in the SES) experienced AEs. SAEs were reported for 22.5%, and SADRs were reported for 5.3%. The most frequently reported AEs were diabetic retinal edema (5.1%), cystoid macular edema (4.0%), concomitant aggravated disease (3.3%), and reduced visual acuity (3.1%). Of a total of 438 AEs, 33.3% had mild intensity, 40.2% had moderate intensity, and 16.9% had severe intensity.

## 4. Discussion

The PACIFIC study provides real-life data on therapy with ranibizumab in local routine clinical practice. It is noteworthy that this study was initiated before the latest publication of the German guidelines [10], which recommended treatment initiation by six monthly intravitreal injections of a VEGF inhibitor. Although the corresponding study data should be carefully interpreted due to certain limitations (selection bias, loss of documentation), five important observations can be made as conclusions about everyday treatment:

(1) Over the last few years, a learning curve has been recognized, which is reflected in key parameters. The median treatment delay was 6 days for pretreated patients and 5 days for treatment-naïve patients in this study. This is notably shorter than in the preceding OCEAN study (conducted between 2011 and 2016), which reported a median treatment delay of approximately 21 days for DME patients [23]. This finding indicates an increased awareness of the importance of early treatment among ophthalmologists in recent years. Success and long-term preservation of visual function depend largely on the function at the start of treatment [24,25];
(2) The intended treatment pattern showed notable differences compared to the actually performed regimen. At baseline, a “fixed” scheme and “pro re nata” were the most commonly used treatment regimens. This would have been in accordance with the DOG guidelines, which recommend a “fixed” scheme for the first 6 months, followed by “pro re nata” treatment [10]. However, in reality, these regimens were only performed for a small percentage of patients. This finding indicates that undertreatment is still an issue for DME patients [26]. Over the course of the observational period, a shift toward a “monitor and extend” regimen was observed based on the statistical derivation. It is possible that due to the study’s defined query and temporal limits, the data led to an interpretation of the study as a “monitor and extend” regimen in many cases, even if not intended by the physician. It seems important to look for unconscious influences and other relevant factors [27]. In the future, attention should be given to realistic agreements to improve the quality of educational discussions and provide informed consent;
(3) Concomitant laser treatment has much less significance than described in clinical studies. Approximately 41–64% of treatment-naïve DME patients (DRCR.net Protocol T from 2012 to 2014) received at least one laser treatment over 2 years, and 23% of pretreated patients and 15% of treatment-naïve patients among the PACIFIC participants underwent concomitant laser treatment. However, it remains unclear whether this difference is ultimately due to the non-interventional nature of the study or to the basic philosophy of a less aggressive combined approach in Europe vs. the USA [28]. Presumably, both influences are likely to play a role if the decision to use a focal-grid laser in addition to anti-VEGF drugs does not follow a systematic algorithm. Although the stability of the achieved visual acuity gain is more relevant for retreatment than retinal morphology is, given the proportions of persistent fluid in DME [11], the strategy may not always be effective because of unclear expectations and perceived non-response. Although combined laser treatment did not show a general benefit in studies, there is indirect evidence for the benefits of targeted supplementary laser treatment over time [29];
(4) The trend toward low treatment numbers was similar between naïve and pretreated patients. Pretreated patients in the PACIFIC study received a mean of 10.6 injections, and treatment-naïve patients received 9.2 injections (among those patients with documentation for at least two years). Thus, patients received notably more injections than in the previous observational OCEAN study (5.5 injections) [30], but still fewer injections than in clinical trials [31,32];
(5) Treatment discontinuation is still a relevant problem in this treatment indication [33]. Aggravated general conditions often cause patients to discontinue treatment. In the PACIFIC study, patients with very high HbA1c values (>9%) were more likely to be discontinued prematurely and experience more AEs. There were no major outliers in terms of insurance or race, but the analyzed cohort was quite homogeneous and of Central European ancestry [34].

The efficacy of the treatment was assessed in representative cohorts. However, by providing real-life data on DME therapy, observational insights from routine clinical practice may lead to a better understanding of the status quo and challenges in patient health care in real-life settings [35,36].

Earlier studies were unable to reveal the considerable deviations between original planning and actual implementation because the planning perspective was not recorded. Specifically, the findings raise the question of whether and to what extent practitioners are aware of the pathways to undertreatment and treatment discontinuation. Overt or hidden resistance, if not the justified and clearly articulated will of the patient, can be relevant. This makes it all the more important that the opportunities for better information—including the relevance of the necessary adherence—are utilized in the future [37].

## 5. Conclusions

In summary, compared with those of previous studies, the present study showed a slight improvement in the real-world treatment of DME patients with regard to treatment delay and the number of injections given during the first year of treatment. However, the current local recommendations [10], suggesting intensive treatment (e.g., comprising six monthly injections followed by further injections based on specific reinjection criteria), were not achieved.

Although regimens clearly define the sequence of control of morphological parameters, fixed re-treatment and determination of the interval for the ‘treat and extend‘ regime, the actual observed behavior after appropriate planning corresponded to completely different patterns. Various factors may contribute to the lack of regular re-treatment while extending, and patients interrupting or terminating the strategy despite the need for intensive treatment.

Deviations in the execution of the plans definitely occur. The search for the reasons and causes can help to improve the treatment of DME in different healthcare settings.

## Figures and Tables

**Table 1:** Demographics and baseline disease characteristics of patients with DME stratified by pretreatment status (FAS).

Parameter	Pretreated N = 458	Treatment-Naïve N = 452
Demographics		
Sex, n (%)		
Male	275 (60.0%)	267 (59.1%)
Female	183 (40.0%)	185 (40.9%)
Age at initial study visit [years], mean (SD)	66.4 (11.7)	66.3 (11.8)
Height [cm], mean (SD)	171.0 (9.1)	170.4 (9.3)
Weight [kg], mean (SD)	86.1 (18.2)	86.4 (17.1)
**Most commonly reported diseases from medical history, n (%)** ^a^		
Diabetes mellitus ^b^	456 (99.6%)	452 (100.0%)
Arterial hypertension ^b^	236 (51.5%)	238 (52.7%)
Hyperlipidemia ^b^	44 (9.6%)	30 (6.6%)
Apoplexy	20 (4.4%)	26 (5.8%)
Myocardial infarct	21 (4.6%)	22 (4.9%)
Renal insufficiency ^b^	25 (5.5%)	18 (4.0%)
**Patients with diabetes mellitus: specification of type, n (%)**		
n	456	452
Type I	50 (11.0%)	46 (10.2%)
Type II	358 (78.5%)	347 (76.8%)
Unknown/Missingc	48 (10.5%)	59 (13.1%)
**Diabetes mellitus: baseline HbA1c value**		
N	189	184
Mean (SD) [mmol/mol]	51.7 (25.7)	49.4 (26.1)
Mean (SD) [%]	6.9 (2.4)	6.7 (2.4)
**Baseline BCVA examination of study eye**		
n	446	443
logMAR, mean (SD)	0.374 (0.317)	0.419 (0.310)
ETDRS letters, mean (SD)	66.3 (15.9)	64.1 (15.5)
**Baseline OCT examination of study eye**		
**Baseline OCT examination performed?, n (%)**		
Yes	313 (68.3%)	379 (83.8%)
No	129 (28.2%)	71 (15.7%)
Unknown/Missing ^c^	16 (3.5%)	2 (0.4%)
**Baseline OCT: central retinal thickness [µm]**		
n	302	369
Mean (SD)	330.0 (108.1)	352.8 (114.1)

^a^ Multiple responses permitted. ^b^ Disease from medical history was explicitly documented as ongoing at baseline for the majority of patients. ^c^ Data were either reported as unknown or not reported (missing). DME: diabetic macular edema, FAS: full analysis set, HbA1c: hemoglobin A1c, N: number of patients in the analysis set, n: number of non-missing observations, SD: standard deviation.

## Data Availability

Patients provided signed informed consent for the publication of their data.

## Abbreviations

The following abbreviations are used in this manuscript:

DME	Diabetic Macular Edema
VEGF	Vascular Endothelial Growth Factor
OCT	Optical Coherence Tomography
BCVA	Best Corrected Visual Acuity
NIS	Non-Interventional Study
SES	Safety Evaluation Set
FAS	Full Analysis Set
AEs	Adverse Events
SAEs	Severe Adverse Events
SADRs	Serious Adverse Drug Reactions
MedDRA	Medical Dictionary for Regulatory Activities
DOG	Deutsche Ophthalmologische Gesellschaft (German Ophthalmological Society)

## Appendix A

**Table A1:** Most common ophthalmological findings (>10%) of the study eye at baseline.

Ophthalmological Findings, n (%) ^a^	Pre-Treated N = 458	Treatment-Naïve N = 452
Subretinal fluid with foveal involvement	142 (31.00%)	170 (37.61%)
Subretinal fluid without foveal involvement	47 (10.26%)	66 (14.60%)
Cystoid fluid inclusion with foveal involvement	265 (57.86%)	274 (60.62%)
Cystoid fluid inclusion without foveal involvement	127 (27.73%)	158 (34.96%)
Diffuse retinal thickening	183 (39.96%)	153 (33.85%)
Ischemic areas	55 (12.01%)	59 (13.05%)
Bleeding or punctate bleeding	275 (60.04%)	288 (63.72%)
Exudate	155 (33.84%)	158 (34.96%)
Intraretinal microvascular anomalies	76 (16.59%)	62 (13.72%)
Proliferative diabetic retinopathy	63 (13.76%)	74 (16.37%)
Cataract	142 (31.00%)	140 (30.97%)

^a^ Multiple responses permitted. N: number of patients in analysis set, n: number of non-missing observations.

## Appendix B

**Table A2:** List of PACIFIC study investigators.

PI	Center	Location
Christos Haritoglou	Gemeinschaftspraxis Profs. Haritoglou, Schultheiß, Klink	Munich
Martin Bechmann	Augenklinik Airport GmbH	Munich Airport
Daniela Erhard	Augenklinik Airport GmbH	Munich Airport
Shervin Mir Mohi Sefat	Augenklinik Airport GmbH	Munich Airport
Gabriele Kuba	Praxis Gabriele Kuba	Munich
Ines Lanzl	Institut Rund ums Auge, I. Lanzl & W. Reich	Prien/Chiemsee
Richard Wertheimer	Augenärzte im Arabellahaus, Richard Wertheimer, Katharina Wittmer	Munich
Udo Heuer	MEC Augenärzte, Udo Heuer	Hamburg
Angela Timm	Augenärztliche Gemeinschaftspraxis. M. Bayer, H. Schneider und A. Timm	Wismar
Bertram Machnik	Praxis Bertram Machnik	Hamburg
Peter Kaupke	Gemeinschaftspraxis Kaupke, Görges, Miebach und Ehrich	Hamburg
Semse Özmen	Augenarztpraxis Gemeinschaftspraxis Wedel	Wedel
Christine Onken	Augenarztpraxis Gemeinschaftspraxis Wedel	Wedel
Soheyl Asadi	Praxis Augenärzte am Weidenbaumsweg	Hamburg
Smbat Berger	Smbat Berger	Bremerhaven
Martin Winter	Praxis Martin Winter	Bremen
Martin Scheffler	Praxis Martin Scheffler	Rhauderfehn
Stephan Kilias	Gemeinschaftspraxis Kilias und Fetter	Hoppegarten
Janek Häntzschel	Gemeinschaftspraxis Häntzschel, Later	Pirna
Linda Later	Gemeinschaftspraxis Häntzschel, Later	Pirna
Marc Marré	Gemeinschaftspraxis Marc Marré	Dresden
Lada Matschke	Praxis Lada Matschke	Neubrandenburg
Kyra Lauritzen	Kyra Lauritzen	Buchholz i.d.N.
Bilal Chamat	Berlin Eye Clinic, Bilal Chamat, FEBO	Berlin
Stefan Heinrich	Praxis Stefan Heinrich	Berlin
Mehrinfar Ben	Gemeinschaftspraxis Dres. Pahlitzsch, Grüngreiff	Berlin
Kathleen Steinberg	Praxis Kathleen Steinberg	Berlin
Jochen Thieme	Jochen Thieme	Berlin
Mohammed O. Ramez	Praxis M. Osman Ramez	Buxtehude
Malek Moubid	Augenarztpraxis Malek Moubid	Buxtehude
Christoph Wehner	Praxis Christoph Wehner, Andreas Meyer-Rößler	Bremervörde
Mayk Steiner	Gemeinschaftspraxis Anke Steiner und Mayk Steiner	Dannenberg
Anke Steiner	Gemeinschaftspraxis Anke Steiner und Mayk Steiner	Dannenberg
Alper Bilgic	Alpha Vision Alper Bilgic, Ahmed Galal	Bremerhaven
Peter Ruokonen	Gemeinschaftspraxis Baecker und Ruokonen	Berlin
Anaelle Laurent	Augenklinik Berlin-Marzahn GmbH	Berlin
Sorin Draghici	Augenarztpraxis Draghici & Kontopoulos	Berlin
Theodoros Kontopoulos	Augenarztpraxis Draghici & Kontopoulos	Berlin
Mirjam Gross	Augenarztpraxis für Gross und Klein	Berlin
Thomas Kube	Praxis Thomas Kube	Bielefeld
Erik Beeke	Visual eins Ärztehaus-Praxis/Klinik	Osnabrück
Susanne Eller-Woywod	Augenärzte Goldmann, Dr.Engels, Dr.Grotheheide, Dr.Eller-Woywod	Gütersloh
Sabine Kaps	Sabine Kaps	Obernkirchen
Nikolai Holak	Nikolai Holak	Salzgitter
Ole Krüger	Augenärzte am Bankplatz Dres. Heinichen, Ahrens, Krüger	Braunschweig
T. Heinichen	Augenärzte am Bankplatz Dres. Heinichen, Ahrens, Krüger	Braunschweig
S. Heinichen	Augenärzte am Bankplatz Dres. Heinichen, Ahrens, Krüger	Braunschweig
M. Ahrens	Augenärzte am Bankplatz Dres. Heinichen, Ahrens, Krüger	Braunschweig
Annette Handstein	Praxis Annette Handstein	Paderborn
Sandra Festag	Sandra Festag	Petershagen
Christof Lenz	Christof Ulrich Lenz	Paderborn
Frank-Christian Nickel	Frank Nickel-Augenärztliche Gemeinschaftspraxis	Peine
Alexander Petzold	Augenzentrum am Johannisplatz, Alexander Petzold	Leipzig
Thomas Hammer	Praxis Thomas Hammer	Halle (Saale)
Gernot Duncker	Gernot Duncker-MVZ Augenheilkunde Mitteldeutschland GmbH	Halle (Saale)
Kerstin Hellmund	Praxis Kerstin Hellmund	Dresden
Matthias Müller-Holz	Überörtliche Gemeinschaftspraxis, Matthias Müller-Holz	Dresden
Tobias Riedel	Überörtliche Gemeinschaftspraxis, Matthias Müller-Holz	Dresden
Regina Matthes	Praxis Regina Matthes	Dresden
Nasser Al-Ashi	Oberlausitz Kliniken gGmbH, Nasser Al-Ashi	Bautzen
Jakub Chmielowski	Praxis Jakub Chmielowski	Plauen
Simo Murovski	Praxis Simo Murovski	Zschopau
Stephan Kretschmar	Praxis Stephan Kretschmar	Bautzen
Dirk Pohlmann	Augenzentrum Osthessen	Fulda
Houcem Ghribi	Houcem Ghribi	Gifhorn
Wolfram Lieschke	Praxis Wolfram Lieschke	Leipzig
Christian Ksinsik	Augenarztpraxis am Glacis	Torgau
Alain de Alba Castilla	Augenarztpraxis am Glacis	Torgau
Jorge Cantu Dibildox	Augenarztpraxis am Glacis	Torgau
Alexander Goldberg	Alexander Goldberg	Coswig
Alexander Stoll	Praxis Alexander Stoll	Chemnitz
Agnes Ute Porstmann	Elbland Augenzentrum am Elblandklinikum-Agnes Ute Porstmann	Radebeul
Gregor Schwert	MVZ Beckum I-Röschinger, Grewe und Schwert	Beckum
Ulrich Thelen	Augenärzte Klosterstraße	Münster
Elisabeth Bator-Banasik	GP Elisabeth Bator-Banasik, Heidi Fischer	Ahaus
Farsad Fanihagh	Gemeinschaftspraxis Ch.-L. Kallmann und F. Fanihagh	Ratingen
Stephan Dunker	Praxis Stephan Dunker	Troisdorf
Maren Unger	Augenzentrum Brühl	Brühl
Frederik Wiegand	Gemeinschaftspraxis Radetzky, Jurek-Becker und Looke, Frederik Wiegand	Neuwied
Hendrik Fuchs	Belenus Augenzentrum Siegen Service GbR	Siegen
Hans-Ulrich Frank	Belenus Augenzentrum Siegen Service GbR	Siegen
Omar Mohamed Alnahrawy	Augencentrum Koblenz, M. Derse, C. Papoulis und Kollegen	Koblenz
Andreas Schmidt	Augenzentrum Andernach Andreas Schmidt, C. Schmidt-Dudziak	Andernach
Christian Abel	Praxis Christian Abel	Trier
Markus Strauß	Praxis Markus Strauß	Saarbrücken
Stefan Pfennigsdorf	Praxis Stefan Pfennigsdorf	Polch
Eduard Berenstein	Augencentrum Koblenz-Höhr-Grenzhausen Derse, Papoulis und Kollegen	Höhr-Grenzhausen
Tatyana Lazarova-Hristova	Praxis Fazil Peru	Frankfurt
Stefan Müller	Praxis Stefan Müller	Neustadt
Martin Rauber	Gemeinschaftspraxis Zuche und Rauber	Saarburg
Andreas Liermann	Andreas Liermann	Neustadt
Isolde Olivas	Praxis Isolde Olivas	Heddenheim
Martin Mundschenk	Praxis Martin Mundschenk	Worms
Gerber Tina	Praxis Tina Gerber	Mutterstadt
Viktor Gossmann	Praxis Viktor Gossmann	Ludwigshafen
Babak Mohammadi	Praxis Babak Mohammadi	Düsseldorf
Hakan Kaymak	Breyer, Kaymak und Klabe Augenchirurgie	Düsseldorf
Johannes Bohnen	LUMEDICO Johannes Bohnen	Düsseldorf
Matthias Grüb	Praxis Matthias Grüb	Breisach
Andrea Wißmann	Praxis Hartmut Karl König	Baden-Baden
Anita Lis-Kowalczyk	Praxis Anita Lis-Kowalczyk	Freudenstadt
Dirk Eberhardt	Gemeinschaftspraxis B. Entenmann und D. Eberhardt	Waldshut-Tiengen
Beatrix Entenmann	Gemeinschaftspraxis B. Entenmann und D. Eberhardt	Waldshut-Tiengen
David Schell	Praxis David Schell	Memmingen
Christian Scherer	Gemeinschaftspraxis Renata Scherer und Christian Scherer	Augsburg
Thilo Schimitzek	Augenklinik Kempten, Thilo Schimitzek	Kempten
Marianne Liedtke-Maier	Augen-Diagnostik-Zentrum Maier, Liedtke-Maier	Hösbach
Othmar Keller	Überörtliche BAG Othmar Keller, Susanne Müller	Herrsching
Jürgen Garus	Praxis Jürgen Garus	Pfaffenhofen an der Ilm
Uwe Schütz	GP Nikolaus Hillenbrand, Uwe Schütz	Ehingen
Christian Schäferhoff	Christian Schäferhoff	Esslingen
Barbara Fuchs-Koelwel	Barbara Fuchs-Koelwel	Regensburg
Klaus Königsreuther	Praxis Klaus Königsreuther	Eckental/Eschenau
Christoph Winkler v. Mohrenfels	Praxis Christoph Winkler v. Mohrenfels	Neutraubling
Magda Rau	Privatklinik Rau	Cham
Thomas Brandl	Praxis Thomas Brandl	Straubing
Georgios Siochos	Praxis Georgios Siochos	Karlsfeld
Janna Harder	Janna Harder	Munich
Stephan Eckert	Gemeinschaftspraxis Kemmerling und Eckert	Sindelfingen
Kamil Weinhold	Praxis Kamil Weinhold	Karlsruhe
Axel Hautzinger	Augenärzte Dres. Reichert und Hautzinger	Frankenthal
Ralph Maria Alles	Praxis Ralph Maria Alles	Saarlouis
Ralf Schmitt	Augen-Zentrum im Medizeum, Ralf Schmitt	Saarbrücken
Susanne Grewing	GP Ralf Grewing und Kollegen	Kaiserslautern
Michael Kusber	Michael Kusber	Bad Arolsen
Björn Feldner	Augenärztliches MVZ Friedländer Weg-Dres. Genée, Feldner und Kolck-Bölling	Göttingen
Stefan Kienzle	Praxis Kienzle, Lojewski und Nolte	Herzberg
Christian Gittner	Christian Gittner	Einbeck
Pia Kirchner	Gemeinschaftspraxis Schröder, Hoerauf, Pia Kirchner	Göttingen
Waldemar Jendritza	Praxis Dres. Jendritza	Ludwigshafen
Annette Brusis	Praxis Annette Brusis	Heppenheim
Harry Domack	Gemeinschaftspraxis Domack, Best und Schmidt	Schweinfurt
Peter Lang	Gemeinschaftspraxis H. Lang, P. Lang, K. Gottschalk	Nürnberg
Judith Becker	Überörtliche BAG Haupt, Spraul, Teuchert und Zorn	Ulm
Carolin Schwamberger	MVZ Augen Rombold, Niederdellmann und Kollegen	Friedberg
Martin Lambert	ÜöBAG Dankwart, Schmidl. Lambert, Schmidt	Rüsselsheim
Frank Koch	Goethe-Universität Frankfurt am Main-Augenklinik	Frankfurt
Svenja Deuchler	Goethe-Universität Frankfurt am Main-Augenklinik	Frankfurt
Ninel Kenikstul	Goethe-Universität Frankfurt am Main-Augenklinik	Frankfurt
Pankaj Singh	Goethe-Universität Frankfurt am Main-Augenklinik	Frankfurt
Katrin Lorenz	Universitätsmedizin der Johannes Gutenberg-Universität Mainz	Mainz
Anna Beck	Universitätsmedizin der Johannes Gutenberg-Universität Mainz	Mainz
Helmut Sachs	Städtisches Klinikum Dresden	Dresden
Merle Schrader	Carl von Ossietzky Universität Oldenburg	Oldenburg
Sabine Aisenbrey	Carl von Ossietzky Universität Oldenburg	Oldenburg
Guido Esper	Carl von Ossietzky Universität Oldenburg	Oldenburg
Menelaos Pipilis	Carl von Ossietzky Universität Oldenburg	Oldenburg
Roland Richter	Augenärzte am Brand 12-Dres. Müller-Richter-Coracas	Mainz
Gregor Eberlein	Klinikum Augsburg AöR-Klinik für Augenheilkunde	Augsburg
Arthur Mueller	Klinikum Augsburg AöR-Klinik für Augenheilkunde	Augsburg
Jürgen Pleines	ZG Zentrum Gesundheit GmbH	Leer
Dieter Hagedorn	ZG Zentrum Gesundheit GmbH	Leer
Klaas Heidemann	ZG Zentrum Gesundheit GmbH	Leer
Nikolaus Lohmann	ZG Zentrum Gesundheit GmbH	Leer
Matthias Meyer	ZG Zentrum Gesundheit GmbH	Leer
Nakisa Amiri	ZG Zentrum Gesundheit GmbH	Leer
Michael Witteborn	ZG Zentrum Gesundheit GmbH	Leer
Hansgeorg Albrechtr	ZG Zentrum Gesundheit GmbH	Leer
Claudia Lanzrath	Berufsausübungsgemeinschaft Zentrum Gesundheit Oldenburg GbR	Oldenburg
Daniela Fromm	Berufsausübungsgemeinschaft Zentrum Gesundheit Oldenburg GbR	Oldenburg
Ramin Khoramnia	Universitäts-Augenklinik Heidelberg	Heidelberg
Gerd Auffarth	Universitäts-Augenklinik Heidelberg	Heidelberg
Dirk Sandner	GWT-TUD GmbH Sandner	Dresden
Matthe Egbert	GWT-TUD GmbH Sandner	Dresden
Hüsnü Berk	St. Elisabeth Krankenhaus Köln-Hohenlind Berk	Cologne
V. Romanou-Papadopoulou	MVZ der Klinik Dardenne GmbH, Vassiliki Romanou-Papadopoulou	Bonn-Bad Godesberg
Hans-Wilhelm Große	MVZ der Klinik Dardenne GmbH, Vassiliki Romanou-Papadopoulou	Bonn-Bad Godesberg
Jasmin Bartling	MVZ der Klinik Dardenne GmbH, Vassiliki Romanou-Papadopoulou	Bonn-Bad Godesberg
Helmut Höh	Dietrich-Bonhoeffer-Klinikum	Neubrandenburg
Mathias Schwanengel	Dietrich-Bonhoeffer-Klinikum	Neubrandenburg
Salvatore Grisanti	Universitätsklinikum Schleswig-Holstein-Klinik für Augenheilkunde	Lübeck
Martin Rudolf	Universitätsklinikum Schleswig-Holstein-Klinik für Augenheilkunde	Lübeck
Matthias Lüke	Universitätsklinikum Schleswig-Holstein-Klinik für Augenheilkunde	Lübeck
Mahdy Ranjbar	Universitätsklinikum Schleswig-Holstein-Klinik für Augenheilkunde	Lübeck
Laurenz Sonnentag	Universitätsklinikum Schleswig-Holstein-Klinik für Augenheilkunde	Lübeck
Thomas Ach	Universitätsklinikum Würzburg Thomas Ach	Würzburg
Jost Hillenkamp	Universitätsklinikum Würzburg Thomas Ach	Würzburg
Kerstin Stange	Augenarztpraxis Stange und Langner	Borna
Andrea Langner	Augenarztpraxis Stange und Langner	Borna
Ulrich Schaudig	Asklepios Kliniken Hamburg GmbH-Asklepios Klinik Barmbek	Hamburg
Kais Al-Samir	Asklepios Kliniken Hamburg GmbH-Asklepios Klinik Barmbek	Hamburg
Birthe Stemplewitz	Asklepios Kliniken Hamburg GmbH-Asklepios Klinik Barmbek	Hamburg
Katinka Westermann-Lammers	Asklepios Kliniken Hamburg GmbH-Asklepios Klinik Barmbek	Hamburg
Gelareh Winter	Asklepios Kliniken Hamburg GmbH-Asklepios Klinik Barmbek	Hamburg
Peter Großkopf	Augenzentrum Hochrhein	Bad Säckingen
Gudrun Papadopoulos	Augenzentrum Hochrhein	Bad Säckingen
Sebastian Tudor	Augenzentrum Hochrhein	Bad Säckingen
Olga Sartorius	Praxis Olga Sartorius	Erkrath
Focke Ziemssen Focke	Universitäts-Augenklinik Tübingen, Focke Ziemssen	Tübingen
Véronique Kitiratschky	Ortenau Klinikum Offenburg-Gengenbach-St. Josefsklinik	Offenburg
Amin Gamael	MVZ der Universitätsmedizin Rostock	Rostock
Joachim Schmidt	Lichtblick MVZ Moers GmbH	Moers
Roland Koch	Lichtblick MVZ Moers GmbH	Moers
Stefanie Schmickler	Augen-Zentrum-Nordwest (MVZ)	Ahaus
Olaf Cartsburg	Augen-Zentrum-Nordwest (MVZ)	Ahaus
Jens Schrecker Jens	Rudolf Virchow Klinikum Glauchau gGmbH	Glauchau
Ute Jüst	Rudolf Virchow Klinikum Glauchau gGmbH	Glauchau
Chris P. Lohmann	Klinikum rechts der Isar-Augenklinik und Poliklinik	Munich
Georg Spital	Augenärzte am St. Franziskus-Hospital	Münster
Frederike Hochhaus	Frederike Hochhaus	Bockhorn
Berthold Seitz	Universität des Saarlandes	Homburg/Saar
Karl-Heinz Emmerich	Klinikum Darmstadt GmbH	Darmstadt-Eberstadt
Andreas Krieb	Klinikum Darmstadt GmbH	Darmstadt-Eberstadt
Monica Lang	Klinikum Darmstadt GmbH	Darmstadt-Eberstadt
Ralf Ungerechts	Klinikum Darmstadt GmbH	Darmstadt-Eberstadt
Christoph Ehlken	Universitätsklinikum Schleswig-Holstein, Campus Kiel	Kiel
Claus von der Burchard	Universitätsklinikum Schleswig-Holstein, Campus Kiel	Kiel
Anna-Maria Kirsch	Universitätsklinikum Schleswig-Holstein, Campus Kiel	Kiel
Ayman Barouni	Ayman Barouni	Hückelhoven
Andrea Wißmann	Praxis Kristin Schubert	Ettlingen
Lena Goldammer	Praxis Lena Goldammer	Celle
Ralf-H. Gerl	Überörtliche BAG Gerl Raesfeld	Raesfeld
Matthias Gerl	Augenklinik Ahaus GmbH & Co. KG	Ahaus
Ralf-H. Gerl	Überörtliche BAG Gerl und Kollegen in Rheine	Rheine
Pascal Hasler	Universitätsspital Basel Augenklinik	Basel
Patrik Kloos	Augenklinik Wil	Wil
Marcel Menke	Kantonsspital Aarau Augenklinik	Aarau
Ioannis Petropoulos	Centre Ophtalmologique de Rive	Geneva
Veronika Vaclavik	Hôpital Cantonal de Fribourg Ophtalmologie	Fribourg
Gabriele Thumann	Hôpitaux Universitaires Genève	Geneva
Fabrizio Branca	Augenzentrum Bahnhof Basel	Basel
Ralf Kiel	Centre neuchâtelois d’ophtalmologie	Neuchatel
Pascal Imesch	Imesch AG	Muri
Daniel Barthelmes	UniversitätsSpital Zürich Augenklinik	Zürich
R.J. Wouters	Oogcentrum Noordholland	Heerhugowaard
L.J. Noordzij	Maasstad Ziekenhuis	Rotterdam
Janneke van Lith	St. Elisabeth Ziekenhuis	Tilburg
V.P.T. Hoppenreijs	Deventer Ziekenhuis	Deventer
Mrs. M. Smeets	Jeroen Bosch Ziekenhuis	‘s-Hertogenbosch
Vicky Boeyden	Ziekenhuis ZorgSaam Zeeuws Vlaanderen	Terneuzen
Thorsten Stolwijk	StolMed Oogklinieken	Bergen op Zoom
J.P. Martinez Ciriano	Oogziekenhuis Rotterdam	Rotterdam

## References

[1] Comyn O., Sivaprasad S., Peto T., Neveu M.M., Holder G.E., Xing W., Bunce C.V., Patel P.J., Egan C.A., Bainbridge J.W. (2014). A randomized trial to assess functional and structural effects of ranibizumab versus laser in diabetic macular edema (the LUCIDATE study). Am. J. Ophthalmol..

[2] Massin P., Bandello F., Garweg J.G., Hansen L.L., Harding S.P., Larsen M., Mitchell P., Sharp D., Wolf-Schnurrbusch U.E.K., Gekkieva M. (2010). Safety and efficacy of ranibizumab in diabetic macular edema (RESOLVE Study): A 12-month, randomized, controlled, double-masked, multicenter phase II study. Diabetes Care.

[3] Nguyen Q.D., Brown D.M., Marcus D.M., Boyer D.S., Patel S., Feiner L., Gibson A., Sy J., Rundle A.C., Hopkins J.J. (2012). Ranibizumab for diabetic macular edema: Results from 2 phase III randomized trials: RISE and RIDE. Ophthalmology.

[4] Ishibashi T., Li X., Koh A., Lai T.Y., Lee F.L., Lee W.K., Ma Z., Ohji M., Tan N., Cha S.B. (2015). The REVEAL Study: Ranibizumab Monotherapy or Combined with Laser versus Laser Monotherapy in Asian Patients with Diabetic Macular Edema. Ophthalmology.

[5] Kodjikian L., Lecleire-Collet A., Dot C., Le Lez M., Baillif S., Erginay A., Souied E., Fourmaux E., Gain P., Ponthieux A. (2021). ETOILE: Real-World Evidence of 24 Months of Ranibizumab 0.5 mg in Patients with Visual Impairment Due to Diabetic Macular Edema. Clin. Ophthalmol..

[6] Sakamoto T., Shimura M., Kitano S., Ohji M., Ogura Y., Yamashita H., Suzaki M., Mori K., Ohashi Y., Yap P.S. (2022). Impact on visual acuity and psychological outcomes of ranibizumab and subsequent treatment for diabetic macular edema in Japan (MERCURY). Graefes Arch. Clin. Exp. Ophthalmol..

[7] Van Aken E., Favreau M., Ramboer E., Denhaerynck K., MacDonald K., Abraham I., Brié H. (2020). Real-World Outcomes in Patients with Diabetic Macular Edema Treated Long Term with Ranibizumab (VISION Study). Clin. Ophthalmol..

[8] Mitchell P., Sheidow T.G., Farah M.E., Mahmood S., Minnella A.M., Eter N., Eldem B., Al-Dhibi H., Macfadden W., Parikh S. (2020). Effectiveness and safety of ranibizumab 0.5 mg in treatment-naïve patients with diabetic macular edema: Results from the real-world global LUMINOUS study. PLoS ONE.

[9] Michl M., Fabianska M., Seeböck P., Sadeghipour A., Haj Najeeb B., Bogunovic H., Schmidt-Erfurth U.M., Gerendas B.S. (2022). Automated quantification of macular fluid in retinal diseases and their response to anti-VEGF therapy. Br. J. Ophthalmol..

[10] (2021). German Society of Ophthalmology (DOG); German Retina Society (RG); Professional Association of Ophthalmologists in Germany (BVA). Statement of the German Ophthalmological Society, the German Retina Society, and the Professional Association of Ophthalmologists in Germany on treatment of diabetic macular edema. Ophthalmologe.

[11] Kalur A., Iyer A.I., Muste J.C., Talcott K.E., Singh R.P. (2023). Impact of retinal fluid in patients with diabetic macular edema treated with anti-VEGF in routine clinical practice. Can. J. Ophthalmol..

[12] Wells J.A., Glassman A.R., Ayala A.R., Jampol L.M., Bressler N.M., Bressler S.B., Brucker A.J., Ferris F.L., Hampton G.R., Jhaveri C. (2016). Aflibercept, Bevacizumab, or Ranibizumab for Diabetic Macular Edema: Two-Year Results from a Comparative Effectiveness Randomized Clinical Trial. Ophthalmology.

[13] Elman M.J., Ayala A., Bressler N.M., Browning D., Flaxel C.J., Glassman A.R., Jampol L.M., Stone T.W., Diabetic Retinopathy Clinical Research Network (2015). Intravitreal Ranibizumab for diabetic macular edema with prompt versus deferred laser treatment: 5-year randomized trial results. Ophthalmology.

[14] Ziemssen F., Schlottman P.G., Lim J.I., Agostini H., Lang G.E., Bandello F. (2016). Initiation of intravitreal aflibercept injection treatment in patients with diabetic macular edema: A review of VIVID-DME and VISTA-DME data. Int. J. Retin. Vitr..

[15] Mitchell P., Bandello F., Schmidt-Erfurth U., Lang G.E., Massin P., Schlingemann R.O., Sutter F., Simader C., Burian G., Gerstner O. (2011). The RESTORE study: Ranibizumab monotherapy or combined with laser versus laser monotherapy for diabetic macular edema. Ophthalmology.

[16] Brown D.M., Schmidt-Erfurth U., Do D.V., Holz F.G., Boyer D.S., Midena E., Heier J.S., Terasaki H., Kaiser P.K., Marcus D.M. (2015). Intravitreal Aflibercept for Diabetic Macular Edema: 100-Week Results From the VISTA and VIVID Studies. Ophthalmology.

[17] Boyer D.S., Yoon Y.H., Belfort R., Bandello F., Maturi R.K., Augustin A.J., Li X.Y., Cui H., Hashad Y., Whitcup S.M. (2014). Three-year, randomized, sham-controlled trial of dexamethasone intravitreal implant in patients with diabetic macular edema. Ophthalmology.

[18] Heng L.Z., Sivaprasad S., Crosby-Nwaobi R., Saihan Z., Karampelas M., Bunce C., Peto T., Hykin P.G. (2016). A prospective randomized controlled clinical trial comparing a combination of repeated intravitreal Ozurdex and macular laser therapy versus macular laser only in center-involving diabetic macular edema (OZLASE study). Br. J. Ophthalmol..

[19] Brown D.M., Nguyen Q.D., Marcus D.M., Boyer D.S., Patel S., Feiner L., Schlottmann P.G., Rundle A.C., Zhang J., Rubio R.G. (2013). Long-term outcomes of ranibizumab therapy for diabetic macular edema: The 36-month results from two phase III trials: RISE and RIDE. Ophthalmology.

[20] Sophie R., Lu N., Campochiaro P.A. (2015). Predictors of Functional and Anatomic Outcomes in Patients with Diabetic Macular Edema Treated with Ranibizumab. Ophthalmology.

[21] Lang G.E., Berta A., Eldem B.M., Simader C., Sharp D., Holz F.G., Sutter F., Gerstner O., Mitchell P., RESTORE Extension Study Group (2013). Two-year safety and efficacy of ranibizumab 0.5 mg in diabetic macular edema: Interim analysis of the RESTORE extension study. Ophthalmology.

[22] Lim J.I., Kim S.J., Bailey S.T., Kovach J.L., Vemulakonda G.A., Ying G.S., Flaxel C.J., American Academy of Ophthalmology Preferred Practice Pattern Retina/Vitreous Committee (2025). Diabetic Retinopathy Preferred Practice Pattern^®^. Ophthalmology.

[23] Ziemssen F., Bertelmann T., Hufenbach U., Scheffler M., Liakopoulos S., Schmitz-Valckenberg S., OCEAN study group (2016). Delayed treatment initiation of more than 2 weeks. Relevance for possible gain of visual acuity after anti-VEGF therapy under real life conditions (interim analysis of the prospective OCEAN study). Ophthalmologe.

[24] Dugel P.U., Hillenkamp J., Sivaprasad S., Vögeler J., Mousseau M., Wenzel A., Margaron P., Hashmonay R., Massin P. (2016). Baseline visual acuity strongly predicts visual acuity gain in patients with diabetic macular edema following anti-vascular endothelial growth factor treatment across trials. Clin. Ophthalmol..

[25] Wells J.A., Glassman A.R., Ayala A.R., Jampol L.M., Aiello L.P., Antoszyk A.N., Arnold-Bush B., Baker C.W., Bressler N.M., Browning D.J. (2015). Aflibercept, bevacizumab, or ranibizumab for diabetic macular edema. N. Engl. J. Med..

[26] Singh R.P., Tabano D., Kuo B.L., LaPrise A., Leng T., Kim E., Hatfield M., Garmo V. (2024). How intravitreal anti-vascular endothelial growth factor initial dosing impacts patient outcomes in diabetic macular oedema. BMC Ophthalmol..

[27] Li S., Pan J., Xu Y., Dai Z., Fang Q. (2025). Exploring the factors influencing the timely intravitreal anti-VEGF treatment in patients with diabetic macular edema: A qualitative interview study using the COM-B model. BMC Health Serv. Res..

[28] Yang X., Cao Y., Cao X., Wang L., Zhang X., Zhang Z., Zai X., Yan Z. (2025). Anti-VEGF monotherapy versus anti-VEGF therapy combined with laser or intravitreal glucocorticoid therapy for diabetic macular edema: A Bayesian network meta-analysis. Diabetes Obes. Metab..

[29] Ziemssen F., Cruess A., Dunger-Baldauf C., Margaron P., Snow H., Strain W.D. (2017). Ranibizumab in diabetic macular edema–a benefit–risk analysis of ranibizumab 0.5 mg PRN versus laser treatment. Eur. Endocrinol..

[30] Ziemssen F., Wachtlin J., Kuehlewein L., Gamulescu M.A., Bertelmann T., Feucht N., Voegeler J., Koch M., Liakopoulos S., Schmitz-Valckenberg S. (2018). Intravitreal Ranibizumab Therapy for Diabetic Macular Edema in Routine Practice: Two-Year Real-Life Data from a Noninterventional, Multicenter Study in Germany. Diabetes Ther. Res. Treat. Educ. Diabetes Relat. Disord..

[31] Wells J.A., Glassman A.R., Jampol L.M., Aiello L.P., Antoszyk A.N., Baker C.W., Bressler N.M., Browning D.J., Connor C.G., Elman M.J. (2016). Association of baseline visual acuity and retinal thickness with 1-Year efficacy of aflibercept, bevacizumab, and ranibizumab for diabetic macular edema. JAMA Ophthalmol..

[32] Payne J.F., Wykoff C.C., Clark W.L., Bruce B.B., Boyer D.S., Brown D.M., TREX-DME Study Group (2017). Randomized trial of treat and extend ranibizumab with and without navigated laser for diabetic macular edema: TREX-DME 1 year outcomes. Ophthalmology.

[33] Peto T., Akerele T., Sagkriotis A., Zappacosta S., Clemens A., Chakravarthy U. (2022). Treatment patterns and persistence rates with anti-vascular endothelial growth factor treatment for diabetic macular edema in the UK: A real-world study. Diabet. Med..

[34] Malhotra N.A., Greenlee T.E., Iyer A.I., Conti T.F., Chen A.X., Singh R.P. (2021). Racial, Ethnic, and Insurance-Based Disparities Upon Initiation of Anti-Vascular Endothelial Growth Factor Therapy for Diabetic Macular Edema in the US. Ophthalmology.

[35] Cantrell R.A., Lum F., Chia Y., Morse L.S., Rich W.L., Salman C.A., Willis J.R. (2020). Treatment Patterns for Diabetic Macular Edema: An Intelligent Research in Sight (IRIS^®^) Registry Analysis. Ophthalmology.

[36] Kuo B.L., Tabano D., Garmo V., Kim E., Leng T., Hatfield M., LaPrise A., Singh R.P. (2024). Long-term Treatment Patterns for Diabetic Macular Edema: Up to 6-Year Follow-up in the IRIS® Registry. Ophthalmol. Retina.

[37] Ziemssen F., Sylvanowicz M., Amoaku W.M., Aslam T., Eldem B., Finger R.P., Gale R.P., Kodjikian L., Korobelnik J.F., Xiaofeng L. (2025). Improving Clinical Management of Diabetic Macular Edema: Insights from a Global Survey of Patients, Healthcare Providers, and Clinic Staff. Ophthalmol. Ther..

